# Resveratrol Modulates Chemosensitisation to 5-FU via β1-Integrin/HIF-1α Axis in CRC Tumor Microenvironment

**DOI:** 10.3390/ijms24054988

**Published:** 2023-03-05

**Authors:** Aranka Brockmueller, Sosmitha Girisa, Ajaikumar B. Kunnumakkara, Mehdi Shakibaei

**Affiliations:** 1 Institute of Anatomy, Faculty of Medicine, Ludwig-Maximilians-University Munich, Pettenkoferstr. 11, D-80336 Munich, Germany; 2 Cancer Biology Laboratory and DBT-AIST International Centre for Translational and Environmental Research (DAICENTER), Department of Biosciences and Bioengineering, Indian Institute of Technology (IIT) Guwahati, Guwahati 781039, India

**Keywords:** CRC, resveratrol, chemosensitisation, 5-FU resistance, β1-integrin, HIF-1α, cancer stem cells, 3D tumor microenvironment

## Abstract

Frequent development of resistance to chemotherapeutic agents such as 5-flourouracil (5-FU) complicates the treatment of advanced colorectal cancer (CRC). Resveratrol is able to utilize β1-integrin receptors, strongly expressed in CRC cells, to transmit and exert anti-carcinogenic signals, but whether it can also utilize these receptors to overcome 5-FU chemoresistance in CRC cells has not yet been investigated. Effects of β1-integrin knockdown on anti-cancer capabilities of resveratrol and 5-FU were investigated in HCT-116 and 5-FU-resistant HCT-116R CRC tumor microenvironment (TME) with 3D-alginate as well as monolayer cultures. Resveratrol increased CRC cell sensitivity to 5-FU by reducing TME-promoted vitality, proliferation, colony formation, invasion tendency and mesenchymal phenotype including pro-migration pseudopodia. Furthermore, resveratrol impaired CRC cells in favor of more effective utilization of 5-FU by down-regulating TME-induced inflammation (NF-kB), vascularisation (VEGF, HIF-1α) and cancer stem cell production (CD44, CD133, ALDH1), while up-regulating apoptosis (caspase-3) that was previously inhibited by TME. These anti-cancer mechanisms of resveratrol were largely abolished by antisense oligonucleotides against β1-integrin (β1-ASO) in both CRC cell lines, indicating the particular importance of β1-integrin receptors for the 5-FU-chemosensitising effect of resveratrol. Lastly, co-immunoprecipitation tests showed that resveratrol targets and modulates the TME-associated β1-integrin/HIF-1α signaling axis in CRC cells. Our results suggest for the first time the utility of the β1-integrin/HIF-1α signaling axis related to chemosensitization and overcoming chemoresistance to 5-FU in CRC cells by resveratrol, underlining its potential supportive applications in CRC treatment.

## 1. Introduction

Colorectal cancer (CRC) is defined as a malignant neoplasm of the colon or rectal epithelium, the treatment of which represents a major medical challenge worldwide [1]. Currently, in the United States of America, approximately 150,000 new cases of CRC are diagnosed annually [1]. In Germany, the number is more than 60,000, and despite numerous early detection measures and techniques, the relative 5-year survival rate in Germany is 63% for men and 65% for women [2]. After diagnosis, most patients receive chemotherapy containing first-line chemotherapeutic agents such as oxaliplatin and 5-flourouracil (5-FU) within the framework of the FOLFOX4 or FOLFOX6 therapy scheme [3,4,5,6].

Oxaliplatin is a platinum derivative chemotherapeutic agent, while 5-FU is one of the main components of the FOLFOX therapy schemes, a synthetic pyrimidine analogue that can be administered intravenously as a prodrug and exerts its effect via fluorinated nucleotides that are incorporated into the patient’s deoxyribonucleic acid (DNA) instead of the pyrimidine nucleoside thymidine [7,8]. It thus inhibits DNA replication and induces cell death in cancer cells [9]. However, in addition to the pronounced aggressiveness of CRC, recognizable by severe metastatic properties of the cells [10], it develops high resistance via activating multiple survival signaling pathways to the mono-target chemotherapeutic agents leading to complicated treatment process with lesser therapeutic outcome or success [6,7,9,11,12]. Moreover, due to the heterogeneity of this disease, it represents a major treatment hurdle, and thus, efforts are being made to explore the exact mechanism or pathways involved in the development of resistance, including dihydropyrimidine dehydrogenase, thymidylate synthase or thymidine phosphorylase signaling pathways [13,14,15,16,17,18,19]. At the same time, biomarkers are being sought that could indicate an unfavorable, chemoresistance-promoting course of FOLFOX therapy at an early stage, with complement compound 3 gene being suggested as an example [12]. Overcoming chemoresistance to mono-target therapies in CRC continues to be intensively explored, and an increasing amount of research is looking at combination treatment of standard anti-cancer drugs and herbal polyphenols [20]. This exciting research strategy is based on a broad, health-promoting spectrum of natural agents such as the blood-glucose-regulative action of chlorella [21] or vitamin C [22] and the anti-oxidative experience of miswak [23] or turmeric-components curcumin and calebin A [24].

Especially for the reduction of CRC-chemoresistance, potential is offered by the secondary plant compound, by the secondary plant compound resveratrol, is a well-known natural polyphenol which can be extracted from various grapes, berries and nuts [25]. Resveratrol has already been shown significant anti-inflammatory [26] and anti-tumor effects [27], especially by modulating the signaling pathways of the important pro-inflammatory nuclear factor ‘kappa-light-chain-enhancer’ of activated B-cells (NF-kB) and NF-kB-related gene cascades [28]. Consistent with the increased number of β1-integrins in CRC cells [29], which function as both cell-survival-protective adhesion and active signaling molecules, resveratrol specifically exploits these β1-integrin receptors in tumor cells by altering their expression pattern and using them as a gateway to transfer its anti-cancer effects and signals into the tumor cells [30]. Interestingly, it was previously described that resveratrol was even able to re-sensitise HCT-116R as well as SW480R CRC cells, which were already resistant to 5-FU by inducing the uptake of this chemotherapeutic agent, thereby overcoming 5-FU-resistance and inducing apoptosis in cancer cells, that would not have been targeted if treated with 5-FU alone [20]. Indeed, the development of chemoresistance is significantly influenced by cancer stem cells (CSC), which have a high renewal and differentiation potential [31].

Moreover, several biomarkers have already been used to detect CSC in HCT116 cells, and it is also known that CSC parameters can be down-regulated by resveratrol treatment in CRC cells [27]. Furthermore, the progression of CRC is also accompanied by an increase in angiogenesis factors, namely hypoxia-induced factors (HIF), as a reaction to hypoxic conditions in the tumor cells, which are responsible for the formation of a new vascular epithelium through the activation of vascular endothelial growth factor (VEGF) cascade [32]. In metastatic CRC, the intratumoral expression of HIF-1α is high as a particularly active subunit, and enables the invasive properties of CRC cells [33,34]. While there is initial evidence of resveratrol-binding HIF-1α restriction in HT-29 [35] and LoVo [36] colon carcinoma cells, thus exploring of the effects of resveratrol on HIF-1α signaling in HCT-116 and 5-FU-resistant HCT-116R CRC cells offers particular scientific interest.

In previous investigations, we have shown the role of β1-integrin receptors in the anti-viability, anti-proliferative and anti-invasive effects of resveratrol [10,30]. However, since it is not yet known whether β1-integrins are also involved in 5-FU-chemosensitisation by resveratrol, we have aimed to address this topic. In addition, we also wanted to shed light on the resveratrol/HIF-1α interaction in CRC cells and to find out whether this connection is influenced by the presence or absence of β1-integrins. Therefore, we chose a pre-clinical, animal-free 3D-alginate tumor microenvironment (TME) culture in vitro and compared the tumor-inhibitory property of resveratrol in two CRC cell lines, HCT-116 and HCT-116R, the later represents a 5-FU-resistant variant of HCT-116 CRC cells.

## 2. Results

### 2.1. Resveratrol Increases 5-FU Sensitivity and Acts Anti-Carcinogenic via ß1-Integrin Receptors in CRC Cells

The ability of resveratrol and its related analogs to increase the susceptibility to the chemotherapeutic agent 5-FU as well as to enhance its effect in CRC cells has already been demonstrated by several research groups worldwide [20,37]. Based on our previous findings that resveratrol employs β1-integrin receptors to transfer its anti-tumor signals into CRC cells [10,30], we hypothesized a connection of resveratrol’s chemosensitisation via these β1-integrin receptors and cells were treated as follows:

First, basal control (CRC cells without additives), TME control (CRC cells in multicellular milieu, without additives), CRC-TME treated with 2 nM 5-FU or 5 µM resveratrol, CRC-TME treated with 2 nM 5-FU and 5 µM resveratrol, CRC-TME treated with 0.5 µM β1-SO (control compound) or 0.5 µM β1-ASO (knockdown compound). Further, CRC-TME treated with 0.5 µM β1-SO or 0.5 µM β1-ASO was supplemented with 2 nM 5-FU or 5 µM resveratrol or with a combination of both substances. Therefore, four different evaluation methods were chosen and their results are described in more detail below.

#### 2.1.1. Reduction of CRC Cell Vitality

**Figure 1:**
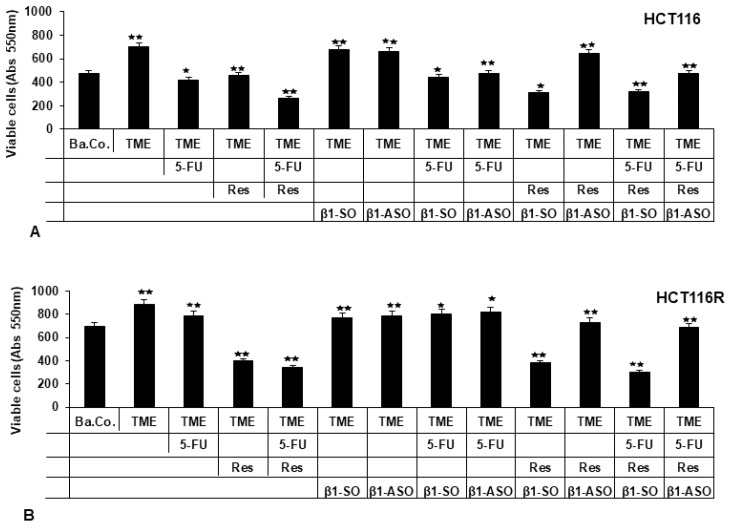
Resveratrol’s viability inhibition and chemosensitisation to 5-FU via β1-integrin receptors in HCT-116/HCT-116R cells shown by MTT assay. *X*-axis: HCT-116 (**A**) and HCT-116R (**B**) cells, grown in alginate, were left untreated as basal control (Ba.Co.), as TME control (TME) or CRC cells in TME were treated with 5-FU (2 nM), resveratrol (5 µM) or β1-SO/β1-ASO (0.5 µM), alone or combined. *Y*-axis: viable CRC cells at 550 nm. Compared to TME control: *p* < 0.05 (⋆) and *p* < 0.01 (⋆⋆).

Compared to the basal control (Ba.Co., without fibroblasts and T-lymphocytes), HCT-116 cells were significantly more viable in the pro-inflammatory TME containing fibroblasts and T-lymphocytes. This remained when β1-SO or β1-ASO were added to the TME, confirming that these substances alone had no relevant effect on HCT-116 cells in the TME. When HCT-116 cells were treated with resveratrol (5 µM), 5-FU (2 nM) or a combination thereof (2 nM 5-FU with 5 µM resveratrol), their survival-capacity decreased markedly and it also decreased when 5-FU was added to HCT-116 cells in the β1-SO-TME or β1-ASO-TME. Resveratrol, however, was able to exert its inhibitory effect in the β1-SO-TME, but not in the β1-ASO-TME (Figure 1A), suggesting that a knockdown of β1-integrin receptors does not affect 5-FU’s effect, but does predominantly restrict the anti-carcinogenic ability of resveratrol in HCT-116 CRC cells.

Then, HCT-116R cells, resistant to chemotherapeutic agent 5-FU, were investigated with the same treatments as HCT-116 cells. Besides the observation that more vital HCT-116R cells than non-resistant HCT-116 cells were measured in general, the main difference was the treatment failure of 5-FU in a TME containing HCT-116R cells and also in β1-SO-TME or β1-ASO-TME with HCT-116R cells. Surprisingly, an addition of resveratrol to TME or β1-SO-TME reduced the viability of HCT-116R cells (Figure 1B), while a knockdown with β1-ASO eliminated the anti-cancer potential and chemosensitising effect of resveratrol in these particularly combative CRC cells.

#### 2.1.2. Reduction of CRC Cell Colony Formation

**Figure 2:**
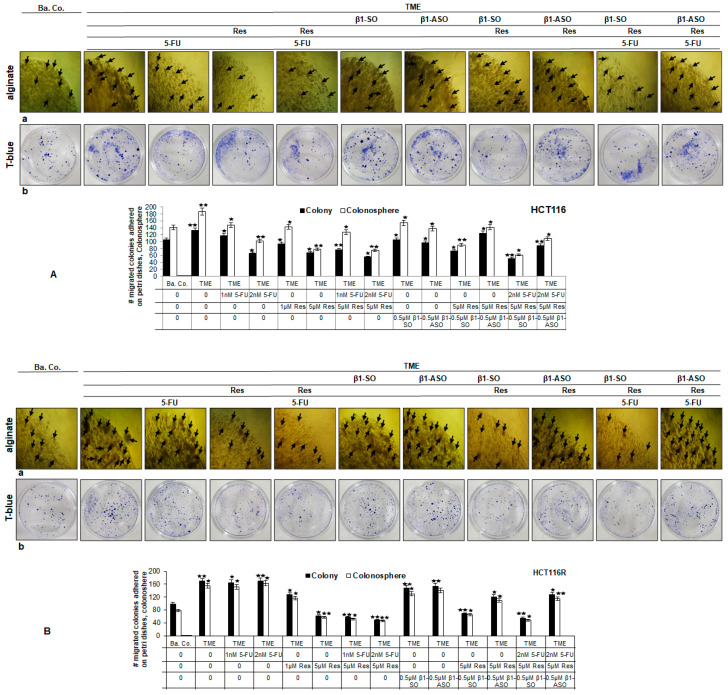
(**A**,**B**) Resveratrol’s colony formation and migration inhibition as well as chemosensitisation to 5-FU via β1-integrin receptors in HCT-116/HCT-116R cells. HCT-116 (**A**) and HCT-116R (**B**) cells in alginate matrix maintained untreated as basal control (Ba.Co.), as TME control or TME-CRC cells were treated with 5-FU (1, 2 nM), resveratrol (1, 5 µM), β1-SO (0.5 µM), β1-ASO (0.5 µM) alone or in combination. CRC-alginate drops were photographed ((**A**,**B**), upper row each) and formed colonies were marked with black arrows. Migrated and settled CRC cell colonies were stained with toluidine blue ((**A**,**B**), lower row each). Statistical evaluation: *p* < 0.05 (⋆) and *p* < 0.01 (⋆⋆), related to TME control. White bars show the colonosphere formed in alginate drops and black bars show the bottom-settled CRC cell colonies.

In TME, HCT-116 cells formed considerably more colonies (black arrows) than in the basal control (Ba.Co.), which was observed to be uninfluenced by an addition of β1-SO or β1-ASO. However, the treatment of these cells with resveratrol, 5-FU, combination of 5-FU and resveratrol, 5-FU and β1-SO or 5-FU and β1-ASO visibly limited the colony formation. Furthermore, resveratrol inhibited proliferative formation of CRC cell colonies in β1-SO-TME but not in β1-ASO-TME (Figure 2A(a); “alginate”). However, when HCT-116R cells were treated with the same agents or additives, the formation of more colonies (black arrows) was noticed compared to HCT-116 cells, while the ineffectiveness of 5-FU in inhibiting colonies was also observed in TME, β1-SO-TME as well as β1-ASO-TME treatments. Interestingly, resveratrol strongly reduced the colony-forming ability of HCT-116R cells in TME as well as in β1-SO, but not in combination with β1-ASO (Figure 2B(a); “alginate”). Overall, this proliferation investigation confirmed the preceding vitality evaluation.

#### 2.1.3. Reduction of CRC Cell Invasion

By comparison with CRC cells in the basal control (Ba.Co.), the number of migrated HCT-116 colonies was higher in TME and also in the β1-SO-TME and β1-ASO-TME. When TME was treated with 5-FU, resveratrol, 5-FU and resveratrol, the invasion capacity of HCT-116 cells was distinctly reduced, similar to an addition of 5-FU to β1-SO-TME or β1-ASO-TME. Moreover, resveratrol-treatment weakened CRC colony settling in TME with β1-SO but not in TME with β1-ASO (Figure 2A(b); “T-blue”). Further extended to 5-FU-resistant CRC cells, smaller but much more migrated colonies settled from HCT-116R cells compared to HCT-116 cells. The same treatments showed the inefficacy of 5-FU to contain HCT-116R invasional property in TME and TME with β1-SO or β1-ASO. Noteworthy, addition of resveratrol to TME or β1-SO-TME averted migration as well as formation of HCT-116R cell colonies, however this effect of resveratrol was not observed in β1-ASO-TME treatment (Figure 2B(b); “T-blue”). These behavioural observations of CRC cell invasion confirmed the previously described MTT and colony formation assays that the β1-integrin receptor might play an important role in the resveratrol-sensitizing effect of 5-FU on CRC cell migration.

#### 2.1.4. Reduction of CRC Cell’s Mesenchymal Phenotype

**Figure 3:**
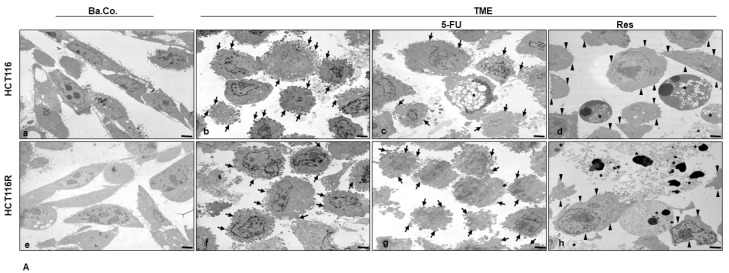

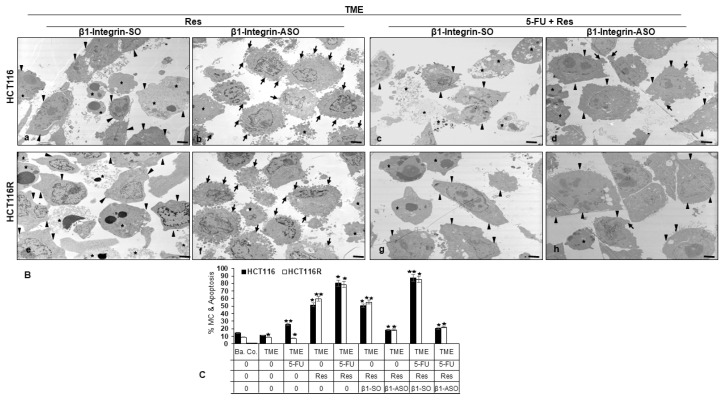
(**A**,**B**) Resveratrol’s phenotype change, apoptosis induction and chemosensitisation to 5-FU via β1-integrin receptors in HCT-116/HCT-116R cells shown by transmission electron microscopy. HCT-116 (**A**(a–d),**B**(a–d)) and HCT-116R (**A**(e–h),**B**(e–h)) in monolayer cultures were cultivated as untreated basal control (Ba.Co.), untreated TME control (TME) or CRC cells in TME were treated with 2 nM 5-FU or 5 µM resveratrol alone or combined with 0.5 µM β1-SO or 0.5 µM β1-ASO and ultrastructural were investigated. Star: apoptosis, arrow: mesenchymal cell extensions, arrowhead: smooth epithelial cell surface. Scale bar 1 µm. (**C**) The statistic diagram illustrates mitochondrial changes (MC) and apoptosis (%) in HCT-116 (black bars) and HCT-116R (white bars) CRC cells with *p* < 0.05 (⋆) and *p* < 0.01 (⋆⋆), compared to TME control.

Both cell types, HCT-116 and HCT-116R, presented in the basal control with elongated cell bodies, planar surface with small pseudopodia on the surface and moderate cell-cell contact, representing a more epithelial morphology (Figure 3A(a,e)). However, in TME, where CRC cells have been influenced pro-inflammatory, HCT-116 as well as HCT-116R cells showed a distinctly mesenchymal shape, in that both the cell bodies and their extensions appeared rounded, developed many thick pseudopodia on the surface with active nucleus and inclined to emigrate (Figure 3A(b,f)). A treatment with 5-FU reduced this TME-induced change in HCT-116 cells without completely restoring the appearance of the basal control (Figure 3A(c)) and occasionally led to the generation of apoptosis, (Figure 3A(c)). In HCT-116R cells, an addition of 5-FU to the TME showed only a slight effect, so that the CRC cells remained morphologically similar to the TME control (Figure 3A(g)). Contrary to this, TME-fueled CRC cells clearly responded to a treatment with resveratrol, as single additive or combined with 5-FU. In both CRC cell lines, HCT-116 and HCT-116R, the cell bodies remained rather roundish to oval, but the round cell extensions needed for migration regressed (Figure 3A(d,h)) and partially re-extended and they had an epithelial shape without pseudopodia in the slightly less aggressive HCT-116 cells in resemblance to the basal control (Figure 3A(d)). Furthermore, the observation of numerous mitochondrial changes and apoptotic bodies, which were even more visible in HCT-116R cells than in HCT-116 cells (Figure 3A(d,h)), was remarkable when CRC cells were treated with resveratrol in combination with 5-FU. To explore the role of β1-integrin in resveratrol’s chemosensitising signaling, HCT-116 and HCT-116R cells in the TME were further subjected to treatment with the knockdown substance β1-ASO or the control substance β1-SO and along with resveratrol alone or combination of resveratrol and 5-FU (Figure 3B). While in β1-SO-TME resveratrol or resveratrol with 5-FU had an almost unrestricted effect of the CRC cells resulting in a smooth epithelial surface (Figure 3B(a,c,e,g)) and initiation of mitochondrial changes as well as apoptosis (Figure 3B(a,c,e,g)), where this effect was severely limited in β1-ASO treatment (Figure 3B(b,d,f,h)). Numerous TME-induced cell extensions remained, especially at combined treatment of β1-ASO and resveratrol (Figure 3B(b,f)), which was very similar to the TME control.

In the combination treatment of β1-ASO with resveratrol and 5-FU, comparatively, strong but somewhat less round cell extensions were observed (Figure 3B(d,h)). As a whole, resveratrol promotes the tendency to an epithelial-like phenotype in CRC cells, making them more susceptible to treatment with the chemotherapeutic 5-FU, and increases apoptosis initiation in HCT-116 and HCT-116R cells, which coincides with outlined results of vitality, proliferation and invasion assays. Summarising this assay, it remains to be noted that (a) HCT-116R cells were indeed predominantly resistant to treatment with 5-FU, (b) resveratrol was effective in both CRC cell types, alone or synergistically in combination with 5-FU and (c) the anti-viable, anti-proliferative, anti-invasive as well as anti-mesenchymal effect of resveratrol was largely cancelled out by the addition of β1-ASO. All in all, the results suggested an anti-carcinogenic and 5-FU-chemosensitising property of resveratrol in CRC cells at least in part via β1-integrin receptors.

### 2.2. HIF-1α Is Involved in Resveratrol-Promoted Chemosensitising CRC Cells to 5-FU

**Figure 4:**
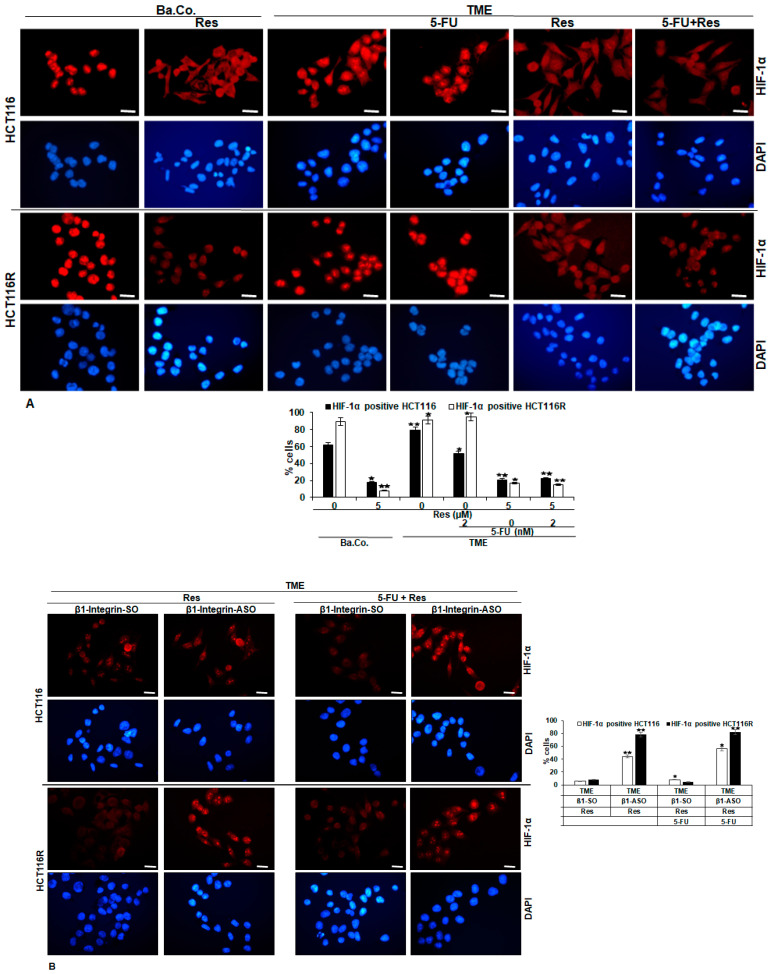
(**A**,**B**) Resveratrol’s enhancement of chemosensitivity to 5-FU by targeting HIF-1α via β1-integrin receptors in HCT-116/HCT-116R cells shown by immunofluorescence microscopy. HCT-116 and HCT-116R were grown on glass coverslips. A basal control was left untreated or treated with 5 µM resveratrol. CRC cells in TME were left untreated or treated with 2 nM 5-FU, 5 µM resveratrol or a combination thereof (**A**). Further, CRC cells in TME were incubated with 0.5 µM β1-SO or 0.5 µM β1-ASO and treated with 5 µM resveratrol alone or 5 µM resveratrol and 2 nM 5-FU (**B**). Thereafter, HCT-116 and HCT-116R were immunolabeled with anti-HIF-1α (red) and DAPI-stained (blue). Magnification ×600, scale bar 30 µm. Statistics: ⋆ *p* < 0.05 and ⋆⋆ *p* < 0.01.

First of all, a slightly different morphology of the two cell lines should be noted, because while HCT-116R cells proliferated more and presented as many small roundish cells with a migratory mesenchymal character, the HCT-116 were in comparison somewhat larger and more epithelially spread out. Both, HCT-116 and HCT-116R were clearly HIF-1α-marked in the basal control (Ba.Co.) containing cell culture medium only. A resveratrol addition down-regulated the HIF-1α expression unambiguously (Figure 4A). This observation was reproduced also in TME composed of floating T-lymphocytes in the cell culture medium, fibroblast monolayers on the well-plate-bottom and CRC cells on glass coverslips. While the HIF-1α expression was strong in the TME control (TME), resveratrol treated HCT-116 and HCT-116R cells showed barely HIF-1α immunolabeling. Noteworthy, a treatment of the CRC cells with the chemotherapeutic agent 5-FU was ineffective in preventing HIF-1α expression, but the combined administration of 5-FU and resveratrol led to a down-regulation of HIF-1α, thus appearing HIF-1α to be a target of resveratrol but not of 5-FU (Figure 4A).

Furthermore, investigations of the mechanistic action of resveratrol in these cell lines confirmed the anti-HIF-1α efficacy of resveratrol even in the presence of the control substance β1-SO alone as well as in the presence of β1-SO and 5-FU in HCT-116 and HCT-116R cells (Figure 4B). Collectively, these results proposed an increased likelihood of HCT-116 and HCT-116R cells responding to 5-FU through resveratrol’s ability to make the CRC cells vulnerable through the β1-integrin/HIF-1α axis.

### 2.3. ß1-Integrin Participated in Resveratrol-Mediated Down-Regulation of NF-kB Activation and Related Gene End Products

**Figure 5:**
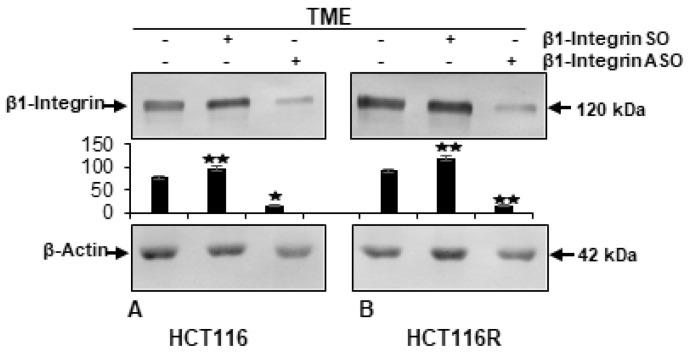
Effectiveness of β1-integrin knockdown by antisense oligonucleotides in HCT-116/HCT-116R cells shown by Western blot analysis. *X*-axis: HCT-116 (**A**) and HCT-116R (**B**) cells in TME-alginate were maintained without treatment or treated with 0.5 µM β1-SO (control) or 0.5 µM β1-ASO (β1-integrin knockdown). Samples were immunoblotted with anti-β1-integrin and further anti-β-actin as loading control. *Y*-axis: densitometric units. Compared to TME control, values were *p* < 0.05 (⋆) and *p* < 0.01 (⋆⋆).

Compatible with previous findings of elevated integrin values in CRC cells [38], a high β1-integrin level has been found in TME in the presence or absence of control β1-SO. But if β1-ASO was added to TME, the level of β1-integrin was down-regulated (Figure 5). All told, β1-integrin knockdown was successfully performed as transient transfection by oligonucleotides listed in Material and Methods.

**Figure 6:**
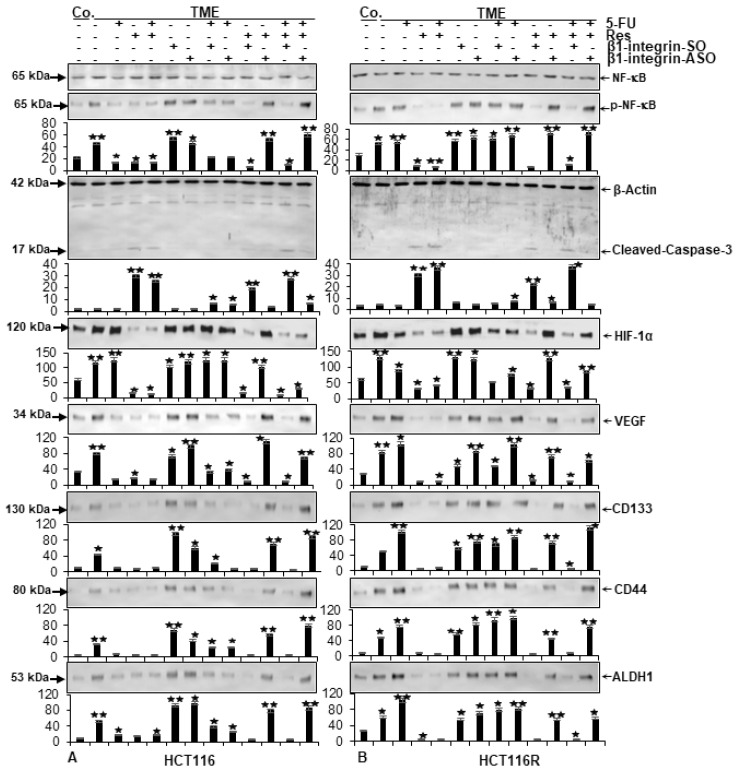
Resveratrol’s reduction of inflammation, vascularisation as well as cancer stemness and elevation of apoptosis via β1-integrin receptors in HCT-116/HCT-116R cells shown by Western blot analysis. *X*-axis: HCT-116 (**A**) and HCT-116R (**B**) cells in alginate drops were left untreated alone (Co.) or in TME, where they were left untreated or were treated with 2 nM 5-FU, 5 µM resveratrol, 0.5 µM β1-SO, 0.5 µM β1-ASO or combinations thereof. Samples were immunoblotted with antibodies against NF-kB (unphosphorylated NF-kB), p-NF-kB (phosphorylated NF-kB), cleaved-caspase-3, HIF-1α, VEGF, CD44, CD133, ALDH1 and β-actin (loading control). *Y*-axis shows densitometric units. Relative to TME control, values were *p* < 0.05 (⋆) and *p* < 0.01 (⋆⋆).

For the extended examination of protein expression level, HCT-116 (Figure 6A) or HCT-116R (Figure 6B) were separated from alginate drops and immunoblotted by SDS-PAGE (Figure 6).

In both CRC cell lines, a sample verification was carried out by the uniform display of β-actin as loading control and the pan-NF-kB was equally represented as a vitality sign in all rehearsals (Figure 6A,B). Following a known, comprehensible signaling chain, the expression of phosphorylated NF-kB (p-NF-kB) as main inflammation parameter, vascularisation factor VEGF as well as CD44, CD133 and ALDH1 as cancer stem cell marker were comparable within each CRC cell line. In HCT-116 cells (Figure 6A), the expression of these markers was higher in TME-cultivated CRC cells than in the basal control and remained high when β1-SO or β1-ASO were added to the TME. With an addition of β1-SO or β1-ASO to 5-FU-treated HCT-116 cells in TME, the expression of parameters mentioned were comparative with the basal control level. Furthermore, a treatment of TME-HCT-116 cells with 5-FU, resveratrol or both agents in combination, significantly down-regulated inflammation (p-NF-kB), vascularisation (VEGF) and cancer stem cell (CD44, CD133, ALDH1) expression. However, remarkably, these anti-CRC effects of resveratrol were reversed by β1-integrin knockdown using β1-ASO, regardless of the presence or absence of 5-FU (Figure 6A). As another representative of vascularisation, HIF-1α level was investigated whereby a decisive difference became apparent. In contrast to resveratrol, 5-FU could not down-regulate HIF-1α and thus could not prevent the initiation of vascularisation. But interestingly, a dual treatment of resveratrol and 5-FU inhibited HIF-1α expression. Resveratrol’s significantly suppressed HIF-1α expression which was also observed remarkably in β1-SO-TME, but not in β1-ASO-TME, regardless of 5-FU’s presence or absence. In a further Western blot analysis on cleaved-caspase-3, this apoptosis marker was up-regulated in all HCT-116 cells in which resveratrol was able to unfold its effect freely, alone or combined with 5-FU. Accordingly, an increased caspase-3 level was noticed in HCT-116 and HCT-116R cells, treated with resveratrol alone or a resveratrol-5-FU combination in TME or β1-SO-TME, but not in a β1-integrin knockdown via β1-ASO in TME (Figure 6A). The key difference in the dynamic observation of 5-FU-resistant HCT-116R cells showed that 5-FU alone or in combination with β1-SO or β1-ASO had no significant anti-inflammatory, anti-vascularising as well as anti-stemness effect (Figure 6B). This was confirmed by the observation where the expression level of all these parameters were comparatively similar to the expression in the TME control of HCT-116R cells (Figure 6B). In contrast, resveratrol alone as well as in combined treatment with 5-FU induced a strong anti-tumor effect against these parameters, both in TME and β1-SO-TME, but not in β1-ASO-TME. In summary, resveratrol reduces inflammation, vascularisation, particularly by inhibiting HIF-1α, and suppresses cancer stem cell formation and increases apoptosis in both HCT-116 and HCT-116R cells, acting chemosensitising and synergistic agent in combination with chemotherapeutic drug, 5-FU at least proportionally via β1-integrin receptors.

### 2.4. Resveratrol Inhibits β1-Integrin/HIF-1a Axis in CRC Cells

**Figure 7:**
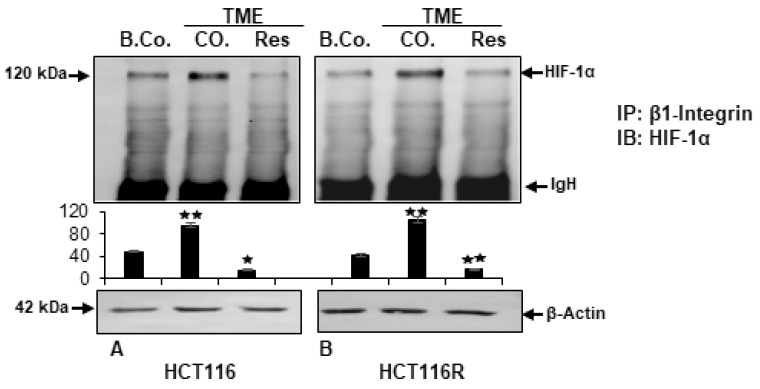
Resveratrol’s attenuation of TME-promoted β1-integrin/HIF-1α axis in HCT-116/HCT-116R cells shown by immunoprecipitation assay. *X*-axis: HCT-116 (**A**) and HCT-116R (**B**) cells in alginate matrix alone (B.Co.) or in TME, were maintained without treatment (CO.) or treated with 5 µM resveratrol (Res). Proteins were immunoprecipitated (IP) with anti-β1-integrin. Each immunoprecipitates was fragmented by SDS-PAGE and immunoblotted (IB) with anti-HIF-1α. Initial samples were provided with anti-β-actin as loading control. *Y*-axis: densitometric units. Values were comparable to reference control, *p* < 0.05 (⋆) and *p* < 0.01 (⋆⋆). IgH means immunoglobulin heavy chain.

The previous results suggested a functional molecular connection between the pathways of β1-integrin and master transcriptional regulator HIF-1α and to investigate this specifically, immunoprecipitation assay was chosen. For this purpose, HCT-116 (Figure 7A) and HCT-116R (Figure 7B) cells were cultured in alginate drops. After 10 days, CRC-samples were obtained, immunoprecipitated with anti-β1-integrin antibody and immunoblotted against HIF-1α to demonstrate the concatenation of both signaling pathways. Consistent with the known β1-integrin expression in the basal control [39], an expression of HIF-1α was analysed by densitometry. In comparison, the expression of HIF-1α in the TME control was markedly increased, consistent with the already shown high β1-integrin expression in Figure 5 and in agreement with the high HIF-1α expression in Figure 4A in both CRC cell lines. Resveratrol impressively suppressed the β1-integrin coupled HIF-1α expression in HCT-116 as well as HCT-116R cells. The uniform β-actin detection served as loading control. Overall, these results suggested for the first time an attenuation of TME-promoted β1-integrin/HIF-1α axis by resveratrol treatment, indicating the intracellular mode of action of resveratrol in inducing anti-tumor effect in CRC cells.

## 3. Discussion

Colorectal cancer management has benefited significantly over the past decade from the development of both target-based therapies and conventional chemotherapeutic substance, such as 5-FU, which have decisively increased the quality of patients’ lives and their life expectancies [40]. However, the performance of these drugs is seriously compromised by the emergence of resistance and recurrence mechanisms which are observed in more than 50% of patients, in routine clinical practice [11]. Therefore, it is the need of the hour to design novel therapeutic compounds for the effective management of such resistant malignancies.

In the past, we demonstrated the importance of β1-integrin receptors in the anti-viability and anti-invasive action of resveratrol, as a natural chemopreventive compound on various CRC cell lines [30]. Pursuing this, the question was whether resveratrol would have a chemosensitising effect to 5-FU via β1-integrin receptors and related signaling pathways on CRC cell lines in a 3D alginate tumor microenvironment. The following core statements could be derived from the results which suggests that resveratrol enhances, at least in part through the use of β1-integrin receptors, (I) intensifies the effectiveness of 5-FU in CRC cells, (II) paves the way for 5-FU efficacy in therapy-resistant CRC cells, (III) triggers the epithelial phenotype, (IV) targets the vascularisation marker HIF-1α and (V) specifically inhibits the association of β1-integrin with HIF-1α in 5-FU-resistant and non-resistant CRC cells.

The results of our study showed that in all the methods used, a significant containment of CRC cells in vitro was observed by treatment with resveratrol in the presence of β1-integrin. Moreover, resveratrol treatment in CRC wells also significantly enhanced the effect of 5-FU when both agents are used in combination. This finding confirms earlier scientific suspicions and hypotheses of a chemosensitising potential of resveratrol in CRC to 5-FU treatment [20,37,41] and it can be assumed that a combination application could also lead to an optimisation of patient’s clinical therapy, for example through accelerated recovery or later during the process of development of resistance. Tumor cells, including CRC cells, are exposed to more severe environmental conditions such as cytokine storms related to inflammatory processes, increased oxidative and endoplasmic reticulum stress as well as hypoxia and changes in the local pH-value [42,43,44]. Such a complex situation was reproduced in our multicellular 3D model, simulating an advanced carcinogenic body situation without animal testing and, as in vivo, by habituation to this environment, the CRC cells become insensitive to environmental influences, including drugs.

A mesenchymal phenotype resulting from epithelial-mesenchymal transition (EMT) as consequence of a pole reversal is a hallmark of aggressive and therapy-resistant tumor cells. During the present study, we observed resveratrol’s ability to change the migratory, mesenchymal and pseudopodia-rich phenotype of CRC cells to a more localised epithelial phenotype thus lowering migratory as well as invasive attendance. In contrast to 5-FU, resveratrol also operated in chemoresistant cells via β1-integrin receptors, thus repressing EMT and paving the way for 5-FU to exert its effect in HCT-116R cells. This could be a potential key component to develop complementary treatment options to control invasive stages of cancer, what is particularly important as more than 50% of patients endure metastases during the course of CRC disease [45] and these often lead to death from organ failure. These findings are in accordance with other studies that have shown that morphological changes and upregulation of intercellular junctions on the surface of cancer cells by resveratrol as a multitargeting component are strongly associated with its anti-malignant and anti-proliferation behaviour in tumor cells [20,46].

Rapidly growing cancerous mass and metastatic processes require a pronounced vascularisation and the angiogenic factor HIF-1α plays a crucial role in this process [47]. As a sensitive parameter, HIF-1α indicates milieu changes in CRC cells, generated in the event of oxygen deficiency, and induces new vessel formation [34] to increase an oxygen supply. In addition, up-regulated αvβ5-integrin or β1-integrin expression as well as NF-kB phosphorylation are associated with a strong HIF-1α increase in CRC cells [48,49]. Plant-derived polyphenols such as curcumin and its analogs could down-regulate HIF-1α expression by interrupting NF-kB phosphorylation in HCT-116 cells [50]. Moreover, HIF-1α has already been suggested as a potential target for resveratrol in various tumor types such as prostate and pancreatic cancers [51,52] or colon carcinoma [35,36], but to the best of our knowledge, the present work is the first study to demonstrate the potential of resveratrol to target HIF-1α in inducing cancer cell chemosensitisation in CRC. Thus, resveratrol could intervene the cancer cascades when the time for local intervention has already passed and the treatment possibilities in patients are limited, thereby increasing the possibilities of general healing chances for patients with advanced CRC.

Each decoding of cancer mechanisms leads to more precise therapy options, reaching the pathologically changed cells more directly and sparing the healthy cells. Thus, it is becoming increasingly interesting to use natural effects of plant ingredients as a co-treatment for various proliferative diseases. For example, inhibition of liver cancer cells by means of oxidative stress and DNA damage induced by safranel, an active component of the spice saffron, has been demonstrated [53]. Furthermore, the flavone quercetin supported significantly the anti-cancer competence of sorafenib (a standard drug against liver cancer) in vitro as well as in vivo by down-regulation of inflammation and up-regulation of apoptosis [54]. Moreover, photosensitive compounds of the plant *Cichorium Pumilum* reduced oxidative stress as well as the activation of estrogen receptors in female Sprague-Dawley rats‘ breast tumors [55]. A crucial factor in chronic progressive diseases, including cancer, is the halting of inflammatory processes. This property has been demonstrated in numerous natural products such as hawthorn from *Crataegus oxyacantha* [56], dandelion from *Taraxacum officinale* [57] and also resveratrol from *Vitis vinifera* [10], that is the focus of this work, in whose detailed effect there is great interest. In establishing the mechanism of action of resveratrol, its effect on tumor suppressor gene p53 was already ruled out in previous study [58]. But interestingly, an in vivo investigation emphasised resveratrol’s significant anti-carcinogenic effect through suppression of oncogenic Kras expression. In this context, the authors describe a 60% inhibition of CRC incidence in mice with activated Kras mutation by supplementing 150 or 300 ppm resveratrol (which in humans would correspond to a dose of 105 and 210 mg) for nine weeks [59]. And now, through this study, we have identified the effect of resveratrol through the β1-integrin signaling pathway as the fulcrum of numerous anti-CRC mechanisms such as proliferation and invasion, and appropriately, showed the importance of β1-integrin receptors in angiogenesis via targeting HIF-1α, and also in chemosensitisation to 5-FU. Especially in view of the fact that β1-integrin is abundant in CRC cells [29], this finding may be helpful in the future for the therapeutical or concomitant use of resveratrol and conventional chemotherapeutics in CRC management.

## 4. Materials and Methods

### 4.1. Trial Substances

The monoclonal antibodies against phospho-p65-NF-kB (#MAB7226), p65-NF-kB (#MAB5078) as well as polyclonal anti-cleaved-caspase-3 (#AF835) were acquired from R&D Systems (Heidelberg, Germany), while monoclonal antibody against β1-integrin (#14-0299-82) was from Thermo Fisher Scientific (Langenselbold, Germany). Monoclonal antibody to β-actin (#A4700), resveratrol, 5-FU, alginate, DAPI, MTT reagent and Fluoromount were purchased at Sigma-Aldrich (Taufkirchen, Germany). Monoclonal anti-HIF-1α (sc-13515), anti-VEGF (sc-7269) and normal mouse IgG were from Santa Cruz (Dallas, TX, USA). Anti-CD44 (ab243894) and anti-CD133 (ab278053), both monoclonal, were from Abcam PLC (Cambridge, UK) and anti-ALDH1 (A248522) was bought as monoclonal antibody from Acris Antibodies GmbH (Herold, Germany). Alkaline phosphatase-linked Western blot antibodies were from EMD Millipore (Schwalbach, Germany) and rhodamine-coupled secondary immunofluorescence antibodies were obtained from Dianova (Hamburg, Germany), while Epon was purchased from Plano (Marburg, Germany). Resveratrol (100 mM in ethanol) and 5-FU (1000 µM in ethanol) were prepared as stock solutions. Both were finally diluted in cell culture medium without exceeding an ethanol concentration of 0.1% during the CRC cell treatment.

### 4.2. Cell Types and Conditioning

HCT-116 (human CRC cells) were obtained from European Collection of Cell Cultures (Salisbury, UK). Chemoresistant tumor cells were produced from these cells by repeated and long-term treatment with 5-FU, and are hereafter referred to as HCT-116R [60]. MRC-5 (human fibroblasts) were from the same institute and additionally, Jurkat cells (human T-lymphocytes) were acquired from Leibniz Institute (Braunschweig, Germany). The preparatory cell culture has already been described in detail [30], as well as the composition of the Dulbecco’s Modified Eagle medium/F-12 cell culture medium from Sigma-Aldrich (Taufkirchen, Germany), which was used with 10% fetal bovine serum (FBS) as growth medium or with 3% FBS as experimental medium [10].

### 4.3. Knockdown of β1-Integrin

Transient transfection was performed, as described earlier [10], with phosphorothioate-specific oligonucleotides from Eurofins MWG Operon (Ebersberg, Germany), incubated in Lipofectin transfection reagent from Invitrogen (Karlsruhe, Germany). The sequences used were β1-integrin-ASO (5′TAGTTGGGGTTGCACTCACACA3′) as antisense oligonucleotide (ASO) and β1-integrin-SO (5′TGTGTGAGTGCAACCCCAACTA3′) as sense oligonucleotide (SO).

### 4.4. Alginate Drop Preparation

Alginate drops were formed, following a method, shown in numerous previous work [30,61]. HCT-116 or HCT-116R were passaged, resuspended in sterile alginate (2% in 0.15M NaCl) and dripped into CaCl2 (100 mM) for polymerization. Afterwards, the resulting CRC-alginate drops were washed with 0.15M NaCl threefold and twofold with cell culture medium (10% FBS). Then, they were incubated in serum-starved cell culture medium containing 3% FBS for 30 min and to start the trial, CRC-alginate drops were transferred with bent tweezers to prepared 12-well-plates.

### 4.5. Carcinogenic Tumor Microenvironment

To illuminate β1-integrin’s role in overcoming chemoresistance through resveratrol, a pro-inflammatory, vivo-near TME was constructed in vitro and treated differently. Therefore, CRC-alginate drops were prepared as described before and placed in experimental 12-well-plates with serum starved cell culture medium (3% FBS), which were changed every second day. The treatments for 10–14 days were as follows: Firstly, a basal control (alginate drops without TME) and a TME control for self-check. Then, concentration-dependent treatments with resveratrol (1, 5 µM) or 5-FU (1, 2 nM) or transfection with β1-ASO/SO (0.5 µM) and finally combinations thereof. Here, TME represents a multicellular, pro-inflammatory composition with a fibroblast-monolayer (MRC-5) on the bottom of the well, floating T-lymphocytes (Jurkat) suspended in the cell culture medium and added CRC-alginate drops. This 3D tumor study model has already been used by our group [10,30,61] as well as, in a similar way, by other research teams [62].

### 4.6. Vitality Assay

Vitality of CRC cells and thus indirect proliferation was detected by detaching the CRC cells from alginate and performing a MTT assay as previously described [30]. Briefly, the CRC-alginate drops were removed from 12-well-plates with bent tweezers and washed in Hank’s salt solution, ensuring that only CRC were examined. Then, CRC cells were extracted from alginate drops by dissolving with sodium citrate. Afterwards, they were washed in Hank’s solution and resuspended in MTT culture medium consisting 3% FBS, but without vitamin C and phenol red. After adding MTT solution and stopping the reaction after 3 h, the Optical Density of samples was measured by Bio-Rad ELISA reader (Munich, Germany) at 550 nm (OD 550).

### 4.7. Proliferation Assay

In order to document the proliferation of HCT-116 and HCT-116R, developed CRC cell colonospheres in alginate drops were photographed with a Zeiss Axiovert 40 CFL (Oberkochen, Germany) phase contrast microscope after 10–14 days of treatment. The images were stored digitally as already done earlier [10,30].

### 4.8. Invasion Assay

CRC cell colonospheres, that had formed in the alginate drops, emigrated from alginate and settled as colonies to the bottom of the 12-well-plates. These colonies were stained with toluidine blue after fixation with Karnovsky solution and stained colonies were manually counted (three independent trials of each treatment), whereas HCT-116 or HCT-116R colonies were certainly distinguishable from fibroblast monolayer, as already published [10].

### 4.9. Immunofluorescence Microscopy

HCT-116 or HCT-116R, grown as monolayer on glass coverslips, were treated 4 h in a modified TME, the detailed procedures were described in our last papers [10,30,61,63], consisting of MRC-5 monolayer on the bottom, floating Jurkat cells in cell culture medium (3% FBS) and small mesh bridges for placing the glass coverslips in 6-well-plates. Afterwards, they were fixed in methanol and prepared for immunofluorescence microscopy as also already been described in detail [30,63,64]. The slides were immunolabeled with a primary antibody against HIF-1α (dilution 1:80), processed with a described secondary antibody (dilution 1:100), DAPI-stained for the assurance of CRC cell vitality and fixed in Fluoromount. Immunofluorescence images were taken with a Leica (Wetzlar, Germany) DM 2000 microscope and related LAS V4.12 software.

### 4.10. Electron Microscopic Evaluation

To investigate ultrastructural changes of CRC cell lines, HCT-116 and HCT-116R were cultivated according to the same experimental set-up as described in the Section 4.9. Afterward, the glass coverslips covered with CRC cells were fixed in Karnovsky solution for 1 h, transferred into a tube with a cell scraper and fixed in osmium tetroxide (OsO4) for 2 h. Then, the dehydration with an ascending series of alcohols and embedding of the cells with Epon were followed as described earlier [31,65]. With a Reichert-Jung Ultracut E (Darmstadt, Germany), samples were prepared, then contrasted with 2% uranyl acetate/lead citrate solution and evaluated with a transmission electron microscope (TEM) 10 from Zeiss (Jena, Germany).

### 4.11. Western Blot Analysis

For immunoblotting, alginate drops with HCT-116 or HCT-116R cells were removed from 12-well-plates with bent tweezers after 10–14 days of treatment. To ensure that Western blot samples contained only CRC cells, CRC-alginate drops were washed as described in the Section 4.6. After dissolving in sodium citrate (55 mM) and freeing from alginate residues, CRC cells were resuspended in lysis mix, centrifuged and the supernatant was frozen at −80 °C. The specimens were processed as described earlier [31,61,63]. We used mentioned primary antibodies and secondary antibodies in dilution 1:10.000 [30]. Samples were blotted with a transblot apparatus from Bio-Rad (Munich, Germany) and densitometric values were evaluated with related “Quantity One” analysis software. β-actin was used as a loading control.

### 4.12. Immunoprecipitation Assay

To investigate the functional relationship between β1-integrin and HIF-1α signaling pathways, CRC cell samples were obtained as described in Section 4.11. Afterwards, they were incubated with 25 µL of normal mouse or rabbit IgG serum and Staphylococcus aureus to preclear, treated with primary antibody against β1-integrin at 4 °C for 2 h and incubated with Staphylococcus aureus at 4 °C for 1 h again according to a proven method [64]. Samples were separated by SDS-PAGE with the explained Western blot technique and apparatus, and anti-HIF-1α antibody was used for this experiment.

### 4.13. Statistical Evaluation

Three independent repetitions were performed from all assays and analyzed by unpaired student’s *t*-test. The results matched by ANOVA (one-way) followed by a post hoc test to compare group parameters. At all outcomes, a *p*-value < 0.05 was considered as statistically significant.

## 5. Conclusions

The current results demonstrate an inhibition of growth, viability and pathological morphological changes and thus both a chemosensitisation of non-5-FU-resistant HCT-116 cells and an overcoming of chemoresistance in 5-FU-resistant HCT-116R cells by treatment with resveratrol along with β1-integrin receptor. For this purpose, resveratrol not only acts as pro-apoptotic (caspase-3) but also induces its effect against inflammation (NF-kB), vascularisation (VEGF), and cancer stem cells (CD44, CD133, ALDH1), and also targets the β1-integrin/HIF-1α axis that is highly pronounced in CRC cells. In summary, resveratrol represents a multifunctional polyphenol that could complement the therapy options of advanced, metastatic or 5-FU-resistant CRC in the future.

## Data Availability

All data are available in the manuscript.
